# “But some were more equal than others:” Exploring inequality at Neolithic Çatalhöyük

**DOI:** 10.1371/journal.pone.0307067

**Published:** 2024-09-06

**Authors:** Katheryn C. Twiss, Amy Bogaard, Scott Haddow, Marco Milella, James S. Taylor, Rena Veropoulidou, Kevin Kay, Christopher J. Knüsel, Christina Tsoraki, Milena Vasić, Jessica Pearson, Gesualdo Busacca, Camilla Mazzucato, Sharon Pochron

**Affiliations:** 1 Department of Anthropology, Stony Brook University, Stony Brook, New York, United States of America; 2 School of Archaeology, University of Oxford, Oxford, United Kingdom; 3 Department of Cross-Cultural and Regional Studies, Copenhagen University, København, Denmark; 4 Department of Physical Anthropology, Institute of Forensic Medicine, University of Bern, Bern, Switzerland; 5 Department of Archaeology, University of York, York, United Kingdom; 6 Hellenic Ministry of Culture, Athens, Greece; 7 School of Archaeology and Ancient History, University of Leicester, Leicester, United Kingdom; 8 CNRS, MC, PACEA UMR 5199, Université de Bordeaux, Pessac, France; 9 Independent Researcher, Berlin, Germany; 10 Department of Archaeology, Classics and Egyptology, University of Liverpool, Liverpool, United Kingdom; 11 Syracuse Academy, Siracusa, Italy; 12 School of Marine and Atmospheric Sciences, Stony Brook University, Stony Brook, New York, United States of America; Austrian Academy of Sciences, AUSTRIA

## Abstract

We explore the ways in which residents of Neolithic Çatalhöyük in Anatolia differentiated themselves as well as the ways in which they did not. We integrate numerous data sets in order to assess patterns of inequality (A) across buildings with contemporaneous occupations, (B) between buildings that did or did not burn at abandonment, and (C) through time. We use Gini coefficients so as to maximize comparability with other studies of inequality in the ancient and modern worlds, discussing the underlying data and our results to clarify and enhance the value of the quantitative analyses. We evaluate whether or not trajectories of inequality align across data sets in order to determine how far success in one realm correlated with success in another. Our results indicate no unified trajectory of inequality through time. We perceive broadly similar access to staple foods, but not to goods less directly related to survival; relatively elevated income inequality during the middle portion of the site’s occupation, plausibly deliberately tamped down; and no evidence for institutionalized or lasting economic or social inequality. These findings shed light on Neolithic social dynamics and also contribute to broader discussions of inequality and the social ramifications of early agropastoralism.

## Introduction

Scholars have long debated whether early agropastoralism was intrinsically associated with the “origins of inequality,” seeking the roots of current cultural practices or problems and largely presenting social developments as the byproducts of sedentism and/or crop agriculture [e.g., 1, 2–5:205–259]. Many now reject any inherent linkage between agriculture and inequality as teleological and materialist [e.g., 6, 7], and there is no longer a consensus that either sedentism or food production automatically entails the rise of inequality greater than exists in hunting and gathering societies [8–13]. It is true that sedentism enables population growth and accumulation of material goods and concentrates resource depletion in limited areas [14–16]. Agriculture sets the stage for economic inequality, as (a) different patches of land have differential productive potential, (b) farmers have built-in incentives to produce surpluses [17, 18], and (c) farmers can live in large communities with heterogenous flows of information and social interaction [e.g., 7]. However, people may ignore or actively avoid taking that stage, and ethnographically documented sedentary food producers do not always have significant levels of inequality [13, 19, 20]. Specific forms of food production may nonetheless bend societies toward not just contemporaneous inequalities but also durable (intergenerationally transmissible) distinctions. Borgerhoff Mulder et al. [21] argue that pastoralism is commonly associated with substantial (if socially unrecognized) distinctions; Gurven et al. [20] tie inequality to reliance on predictable and monopolizable resources that are available in limited quantities; Smith and Codding [22] correlate institutionalized inequality with reliance on spatiotemporally clumped resources (wild or domestic); Shenk et al. [23: 80] claim that intensive (meaning plow and/or irrigation) farming is necessary, if not sufficient, for persistent inequality to emerge.

Many scholars have investigated the extents and possible forms of inequality in early agricultural societies. Some researchers have focused on potential economic disparities [11, 24–27, see also, 28]; some on political distinctions [29–32]; some on ritual differentiations [33:18, 34:114, 35, 36]; and relatively few on multiple dimensions of asymmetry [37, 38]. Motivated, in part, by a desire to understand the development of institutionalized hierarchy and of economic complexity, archaeologists working around the globe debate whether various forms of food production propelled non-age-or-sex-based inequities and how any such inequities may have manifested socially [39–42].

### Inequality among early southwest Asian agropastoralists

Inequality existed in Neolithic southwest Asia. Within any early farming village or town, at any moment in time, some residents were children and some adults; some were healthy and skilled, and some unwell or inept. In any given year some fields would produce more than others, and some animals would thrive while others fell ill or simply grew old. Neolithic people participated differentially in society, and productive capacities varied between households. Scholars of early agropastoralism debate, however, whether unequal production coexisted with inequalities in other spheres of life, and whether economic or social distinctions were temporary or transferrable across generations [e.g., 11, 25–32, 37–41].

Over the decades researchers have inferred a wide variety of social structures and interpersonal relationships among the sedentary villagers and townspeople of Neolithic southwest Asia (ca. 10,200–6000 bp/9700-5300 cal BCE, but here and throughout the paper we speak only of eras and areas possessing the full Neolithic “package” of domesticated animals as well as plants). It is nonetheless clear that during the Neolithic, large, densely populated settlements arose, whose hundreds—in the largest sites, perhaps even thousands—of inhabitants farmed wheat, barley, and legumes, herded caprines and cattle, built complex and sometimes multistory stone and mudbrick structures, traded across large distances, and interacted regularly with sizable networks of kin, neighbors, friends, and relatively distant acquaintances.

Scholars of early southwest Asian agropastoralism have argued for Neolithic hierarchical distinctions [29, 41, 43, 44], for egalitarianism [broadly defined, e.g. 6:72, 25:163], and for cultures in which social mechanisms suppress distinctions that would naturally arise [45–50]. Due in part to a tendency to base such analyses on relatively few lines of evidence (commonly one or two data sets and their architectural contexts [e.g., 51–53]) and in part to the difficulty of acquiring data sets large, detailed, and chronologically controlled enough to shed light on *patterns* of inequality within settlements rather than anecdotal evidence, we still largely lack studies that a) evaluate multiple possible forms of inequality and b) examine both synchronic and diachronic distinctions.

Here we attempt to fill this gap by deploying detailed artifactual, ecofactual, mortuary, and architectural data sets from the Early Ceramic Neolithic site of Çatalhöyük in central Anatolia in order to better understand Neolithic social organization. We compare and contrast a wide array of assets across houses in order to evaluate differences in subsistence income, productive capacity, symbolic elaboration, and “costly” or “prestige” personal goods. We assess whether inferred household income was directly or inversely associated with political or symbolic wealth, and examine evidence for the durability of house differences. We consider the extent to which ancient house abandonment practices shape modern perceptions of inequality, and evaluate whether levels or forms of inequality changed over the course of the site’s occupation.

We use differences in material culture as proxies for differences in social position, acknowledging the necessary caveats as we discuss potential distinctions between productive capacities, staple assets, and relational positioning [e.g., 37, 54:17, 55, 56]. We calculate Gini coefficients for each of the data sets we examine in order to maximize comparability with other studies of inequality in the ancient and modern worlds [39, 57–59], but view them as conversation-starters rather than final conclusions. We stress that whereas in archaeology “inequality” has often been used to refer to hierarchical differentiation, our quantitative analyses simply reveal the extent of dissimilarity in different data sets at Çatalhöyük. The dissimilarities thus discovered cannot necessarily be equated with a hierarchical social order, and they certainly cannot be taken as proof of institutionalized inequities.

The goal of this paper is thus to explore the ways in which Çatalhöyük people differentiated themselves as well as the ways in which they did not. Doing so enables us not just to shed light on Neolithic social dynamics but also to contribute to broader discussions of inequality and the social ramifications of early agropastoralism.

### Neolithic Çatalhöyük

**Fig 1:**
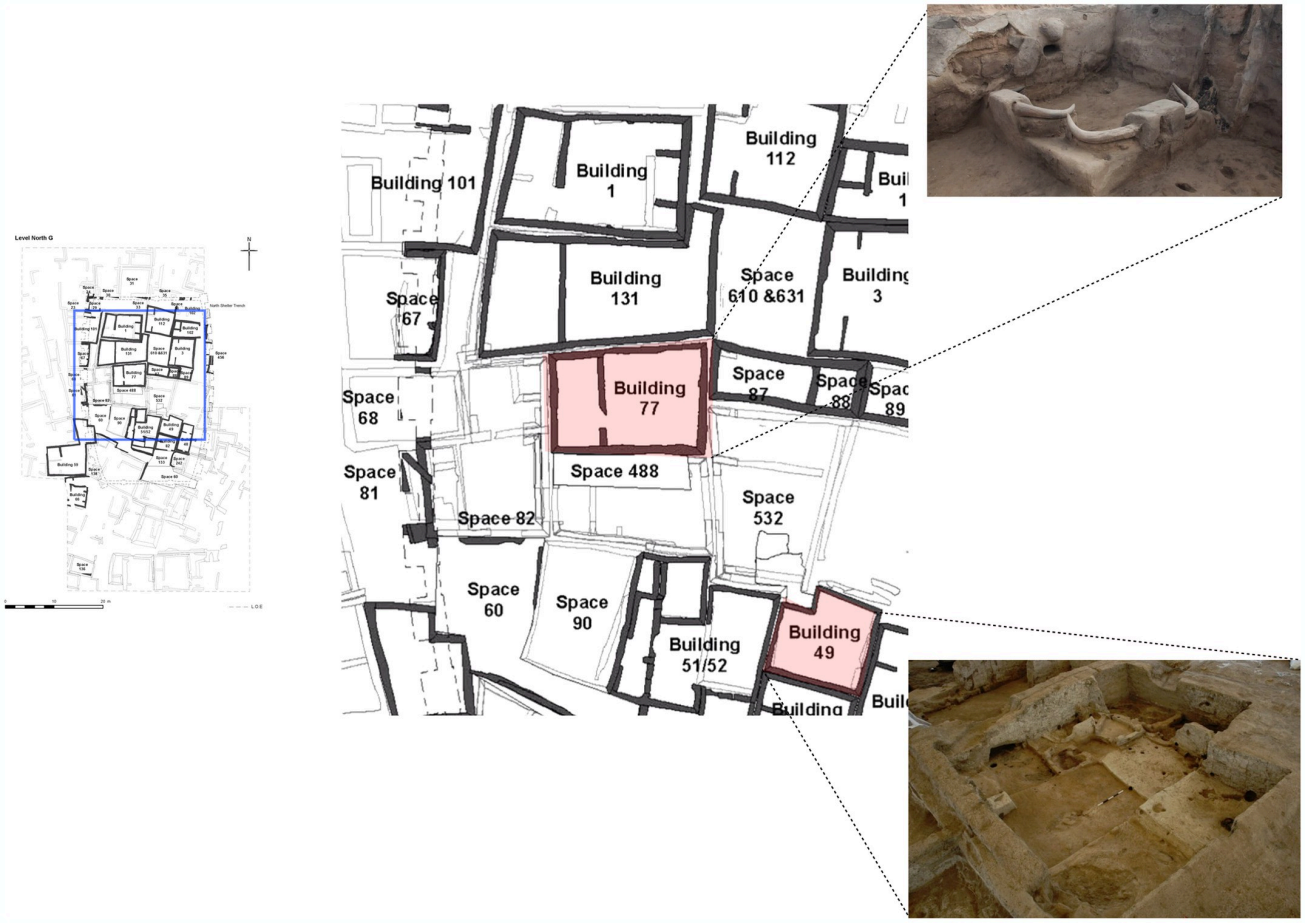
Plan of Çatalhöyük North Area, with photos of select buildings during excavation. Photographer Jason Quinlan; copyright Çatalhöyük Research Project.

During the early and middle 7^th^ millennium BCE, the residents of Neolithic Çatalhöyük in central Anatolia occupied a settlement like none other on the Konya Plain [60–63]. Above fields, rivulets, and scattered trees, closely packed rectangular mudbrick structures (Fig 1) housed townspeople who lived in clusters so tightly packed that many people crossed their neighbors’ abutting roofs in order to descend via ladder into their doorless homes. The number of residents fluctuated through time: recent estimates range from several hundred to perhaps a few thousand at peak occupation [64, 65], with perhaps seasonal as well as longer-term shifts in numbers. All buildings show signs of residential occupation, and three decades of excavation [66–71] have revealed no communal architecture or generally accessible spaces predating the topmost layers of the mound [61, although see, 72, 73]. It is generally agreed that life at Çatalhöyük took place in, around, and above the homologous residential structures that we here term houses.

These houses were individually built and contain hearths, platforms, and storage spaces. Inside them we find evidence for activities ranging from stone working to grain grinding to ritual interment [63, 74–82]. Animal bones embedded in walls, benches, and posts as well as stones and bones placed in foundation and commemorative deposits testify to a domestic sphere saturated with symbolism and ritual [80, 83–86]. Architectural, artifactual, and ecofactual data reveal differential use of indoor space, with cooking and stoneworking typically conducted in buildings’ southern halves and symbolic installations placed near their northern walls. Burials are typically found under houses’ northern floors, although extremely young infants are more often found in southern areas and side rooms [82]. Indeed, the regularity with which buildings display similar layouts and uses of space is remarkable. Idiosyncrasies certainly exist, but with 197 buildings partially or wholly uncovered (144 by Mellaart’s team; 53 in the modern era) we see only modest deviations from what must have been strong cultural norms [87–89]. Buildings underwent repeated modifications during their occupations, which typically lasted between 50 and 100 years [90–92]. Most buildings were thoroughly cleaned out at abandonment, their installations dismantled and their floors carefully swept. A handful of buildings burned without being emptied, however: debate continues as to the intentionality of their incineration, and thus as to the extent to which their contents reflect daily life inside the structure [83, 93–96].

### Houses as a unit of analysis for studying inequality at Çatalhöyük

We argue here—as have others at Çatalhöyük and other southwest Asian Neolithic sites [50, 97–103]—that using buildings (“houses”) as the primary unit of analysis for comparing and contrasting evidence from across the site is a valid approach. Nevertheless, these structures likely did not function wholly autonomously [e.g., 104–106]. Ethnographically and ethnohistorically documented farming households rely on and socialize extensively with each other [e.g., 107, 108], and archaeologists have inferred non-coresidential social groups at multiple Neolithic sites in the southern Levant as well as at Çatalhöyük itself. Hodder & Pels [109] and Düring [52, 110, 111] posit that at Çatalhöyük neighborhoods may have been foundational elements of social organization; Kuijt [112] suggests that multifamily Houses (*sensu* House Societies) organized both social and ritual life; Mills [113] and Rosenberg and Rocek [45] argue that sodalities were important. We nonetheless consider individual buildings to have been “the key component of the social fabric and… the main and enduring principle of social organization,” as supra-household groupings were relatively fluid and interdigitating rather than fixed and distinct [88:8].

Evidence cited for supra-household social groups elsewhere in the southwest Asian agropastoral Neolithic includes anthropomorphic statuary [114]; stylized plastered skulls [114]; possible cult objects found inside houses [114]; individuals interred in “trash” (middens) outside houses [114]; stone masks [45]; remains of feasts [47, 115]; crop isotopes that are broadly similar across a site [116]; and non-residential structures, spaces or monuments inferred to have been built for multi-household ceremonies or rituals [e.g., 7, 45, 102, 114, 117–119]. Few of these lines of evidence exist at Neolithic Çatalhöyük. Here, the conversations around supra-household social life have centered around (a) inferred relationships between structures [e.g., 52, 88, 111, 112, 120, 121]; (b) inferred feasting remains [76, 122]; (c) differential numbers of burials and architectural elements across houses [52, 109–112, 123, 124]; (d) wall paintings depicting group hunts and people who might be wearing costumes [45, 113]; (e) shared technologies [e.g., 125:14, 126]; and (f) the sheer size of the settlement [72] and its location in a variably productive landscape, which plausibly caused residents to have to coordinate agricultural production, in combination with botanical remains indicating intensive crop management (consistent with cooperative labor) and variably public crop processing [116].

From our perspective, the balance of evidence at Çatalhöyük does not point to supra-household social units with durable, unambiguous spatial “footprints” [contra 112, 127] that would allow quantitative comparison between such communities. Rather, such larger communities appear shifting, contextual, rooted in particular practices and interleaving in space. Architectural connections between houses are scattered both spatially and chronologically, and take no consistent form. We perceive no patterned repetition as might suggest systematized social relationships involving multiple houses’ residents. Nor do discrete artifactual assemblages or sets of features (analogous to archaeological ‘cultures’, *sensu* Childe [1]) recur across houses, as we might expect if households consisted of multiple dispersed residences with inhabitants of different social or economic statuses, as in classic historical “house societies” [e.g., 128–130]. Stable isotope analyses of house “groups” reveal no significant dietary clustering [131]. Households surely collaborated agriculturally [116, 132], but extensive investigation has not enabled identification of specific cooperative units or even their likely scale. Some of the site’s zooarchaeological deposits and stable isotope values reflect food sharing at scales larger than the individual household [76, 133, 134], but participant numbers are estimates at best and participant identities pure guesswork. Mazzucato identified clusters of buildings that shared a larger amount of material culture and thus were probably related; she does not, however, suggest that these groups superseded individual houses’ economic or social agency, stressing instead that her data indicate “a marked…site-wide shared community identity” [88:21].

**Fig 2:**
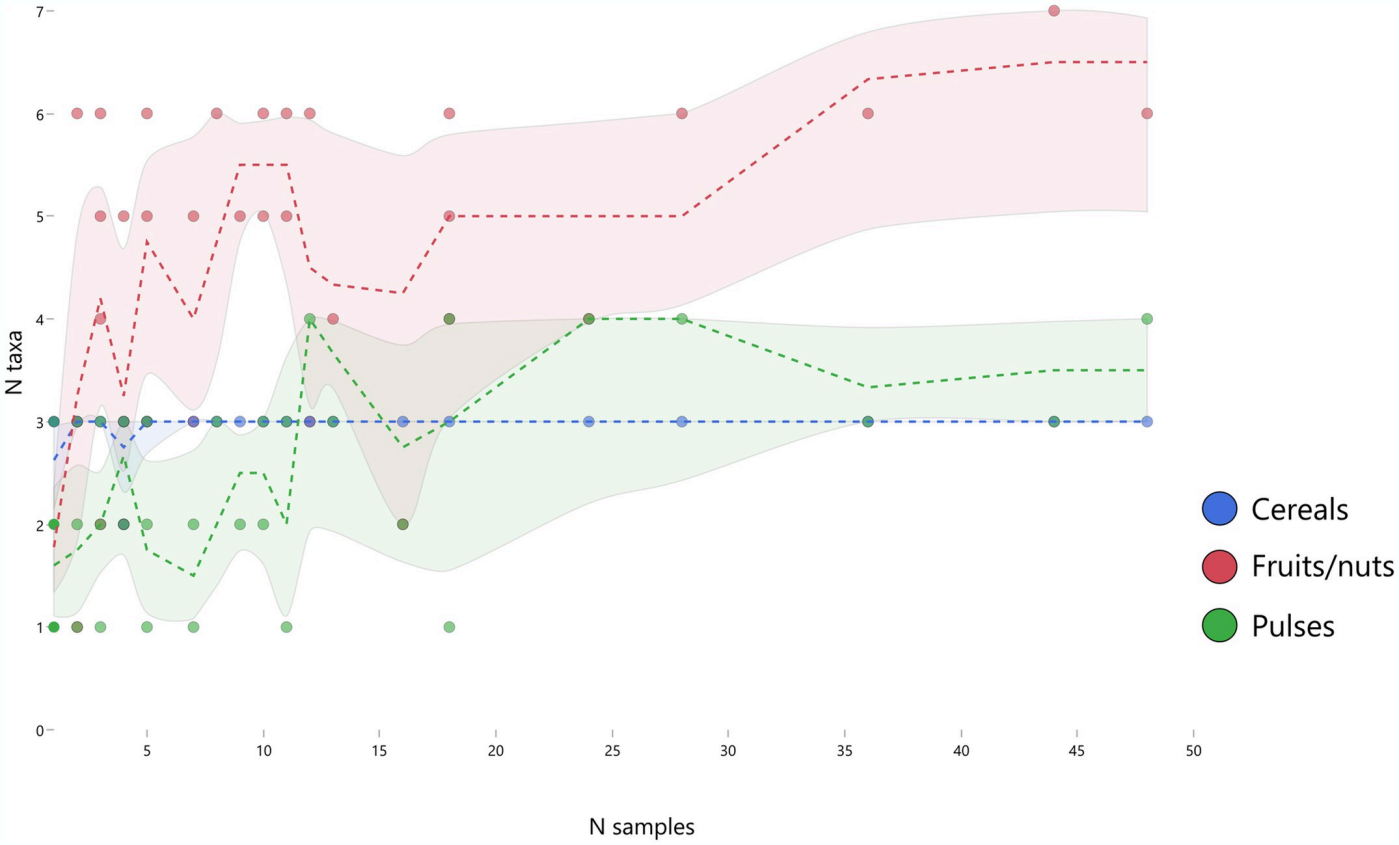
The number of plant taxa identified in buildings at Çatalhöyük. Forty-eight buildings are represented, arranged along the x-axis according to their number of analyzed macrobotanical samples.

Our understanding of houses’ position as the basal socioeconomic units of society is enriched by botanical data from houses across the settlement (S1 Table). All of Çatalhöyük’s structures contain botanical deposits that reflect in situ use such as fire installations and adjacent “dirty” floors [132]. Plant taxa consumed at Çatalhöyük include cereals, pulses and other non-canonical crops, and gathered resources: small-seeded mustard, nuts like acorn and almond, and fruits like hackberry (which survives without charring due to its carbonate-rich shell). Across the settlement and through time, individual houses parallel each other in their suites of staples. Fig 2 shows that most (40/48) buildings across the site contain evidence for cereals, pulses and fruit/nut taxa, but all buildings represented by at least five samples contain all three categories. Moreover, all of the buildings represented by at least five samples contain evidence for glume wheat, barley, and free-threshing wheat, plus one to four pulses and two to seven fruit/nut taxa. All reasonably-well-sampled buildings thus have yielded evidence for a similar variety of cereals, pulses and fruits/nuts. S2 Table, which examines high-density concentrations of plant material found in burned buildings, corroborates that the occupants of most if not all buildings enjoyed equitable access to and use of cereals, pulses and other food plants.

This pattern could, of course, be attributable to resource pooling: it is inconsistent with some structures having atypical purposes or uses (e.g., sodality ‘ritual buildings’), but not with multi-residential economic units. The houses do not, however, all contain the same plants: they have similar *diversities* of taxa, not identical lists of species. There are clear differences in the selection of particular taxa among buildings, particularly amongst the glume wheats, pulses and fruits/nuts. Some of these differences appear to distinguish plant use between the North and South Areas through the middle of the site occupation sequence (e.g. new type in the North, emmer in the South; lentil in the North, pea in the South), but this is a question of emphasis/degree, not of exclusive access [51, 132]. For example, “new type” glume wheat was present at low levels relative to emmer throughout the earlier Neolithic in the South Area. The broad difference between neighborhoods appears to have been a result of conscious decisions to experiment with minor crops, such as “new type” glume wheat in the North and pea in the South. The permeation of such innovations across buildings and areas of the site also speaks to general equality of access to food plant resources. The similarity and redundancy—but not identicality—of house plant assemblages is thus a strong argument in favor of household units. Recovery of in situ botanical remains from indoor storage installations demonstrates further that staple goods were retained in, and therefore presumably controlled by, the houses.

We thus conclude that at Çatalhöyük the individual house is the archaeologically clearest and most appropriate unit of analysis. As noted above, such houses were surely not fully autonomous, but no data suggest that multi-residential bonds superseded co-residential ones. Amid the “dispersed overlapping mosaic of relationships” [121:153] that characterized life at Neolithic Çatalhöyük, those located in and around the house shaped people’s lives economically, socially, and ritually.

Our analyses assume that each house was associated with a basal socioeconomic unit (not necessarily a nuclear family [135–137]) and that its attributes—size, elaboration, contents—reflect traits of that unit although not necessarily *only* that unit’s traits. Such assumptions likewise underpin previous analyses of social and economic inequality at Neolithic Çatalhöyük. Researchers such as Hodder [121], Wright [53], Bogaard et al. [75], Conolly [138], Mazzucato [88], Love [139] and Düring [52, 110, 111], have compared and contrasted structures’ artifactual, ecofactual, or mortuary contents in order to explore potential economic, social, and/or ritual distinctions. Conclusions have varied considerably: “Social inequality is suggested by the sizes of buildings, equipment, and burial gifts…” wrote James Mellaart after he finished his years of excavation at Neolithic Çatalhöyük in central Anatolia [66:225], whereas Ian Hodder [89:5] describes a “fierce egalitarianism.” Asouti [140:87] infers an “essentially egalitarian society… [but with] a certain measure of asymmetry and competition in relations between individuals and households”, while Rosenberg and Rocek [45:26] perceive “inequality and sodality-related hierarchy constrained by a communal egalitarian ethos” and Der and Issavi [87:201] argue for “heterarchical house-based social organization.” The extent and form(s) of inequality at Çatalhöyük thus remain unresolved; some researchers find social inequality where others find no or limited social disparity, while others describe mechanisms that acted to reduce such disparities.

In this study we examine inequality at the settlement from three different angles. First, we evaluate the equality of remains associated with houses inhabited during a single phase of the site’s occupation. We then assess whether perceptions of inequality are skewed by differential modes of abandonment, exploring levels of inequality across houses that burned as opposed to those that did not. Finally, we investigate whether patterns of differentiation changed through time.

## Materials and methods

Our study diverges from previous analyses in both approach and scale. To begin with, we do not use the stratigraphic chronology established by Mellaart in the 1960s [cf. 52, 111, 112]. Single Mellaart occupation phases include structures now believed to have been constructed at different times and occupied for varying durations: many buildings were left open after abandonment and before infilling, so houses and spaces that appear contemporaneous in Mellaart plans were, *contra* several previous analyses, not all in use at the same time [72, 141]. Mellaart’s building plans also omit relevant features, mislocate walls by up to 2.5m, and present houses as static entities rather than as the multiphasic changing structures we now know them to have been [124]. We therefore use “Hodder Levels,” which use recently-excavated stratigraphic and architectural relationships to establish a comparatively fine-grained relative chronology [142]. Bayesian analysis of the site’s ^14^C dates is still underway, but we can anchor Hodder levels in larger site occupation phases with assigned radiocarbon dates (Table 1).

**Table 1:** Çatalhöyük chronology: Levels and occupational phases. After Farid 2014; Mazzucato 2019.

Çatalhöyük occupation phase	Hodder Level		Cal BCE *(circa)*
	*South Area*	*North Area*	
**Late**	South P-T	North H-J	6500–6300
**Middle**	South M-O	North F, G	6700–6500
**Early**	South G-L	n/a	7100–6700

In order to ensure that our analyses rest on comparable and reliable datasets, we include only buildings that are at least 75% excavated, with architecture and features verified by the modern Çatalhöyük Research Project (ÇHRP). By “fully excavated” we mean that not only have all architecture and building contents been recovered, but also all subfloor deposits. To maximize direct comparability of data, we include only buildings from the Early, Middle, and Late phases of the site’s occupation, as Çatalhöyük’s Final Neolithic/Chalcolithic occupations were excavated and analyzed by teams and using protocols that largely but not completely overlapped with those used throughout the earlier phases [see 61, 143–145]. Twenty buildings meet these requirements (Table 2, S3 Table).

**Table 2:** Buildings included in this study. For a complete tally of their features and contents, including data sets not discussed in the main body of this paper, please see S3 Table.

					"Income"	Productive capacity		Symbolic/Relational Assets							"Costly" or prestige portable goods			
Occupation phase	Building	Level	% of building that was excavated	Burned building?	Side room m^2^	# of grinding tools with use faces	fixed grinding installations = area of use face in cm^2^	Paintings	Faunal Installations (in situ)	Faunal special deposits	Burial MNIs (primary and secondary)	# of primary individuals	# of secondary individuals	Grave goods (intentionally placed)	Exotic shells	# of exotic shell sources	# of exotic stone beads	# of non-local stone material types
Early	**2**	South K	75		5.1	0	0	2	0	7	0	0	0	0	8	2	0	0
	**17**	South K	75		9.1	0	0	31	0	0	25	25	0	57	16	2	0	1
	**160**	South K	100		6.7	0	0	2	0	17	4	4	0	8	1	1	0	1
	**43**	South L	75		4.9	0	0	3	0	0	9	9	0	8	3	1	0	1
Middle	**119**	North F	75		6	0	0	2	0	0	0	0	0	0	0	0	0	3
	**132**	North F	75		*n/a*	0	0	3	0	0	10	10	0	29	6	2	1	1
	**1**	North G	100	y	9.8	0	0	21	2	15	60	52	8	72	85	2	3	2
	**3**	North G	100		6.3	0	0	15	2	30	8	8	0	20	6	2	0	2
	**49**	North G	100		2.3	0	0	31	0	25	15	15	0	38	54	2	0	3
	**51**	North G	100		0	0	0	0	0	0	0	0	0	0	3	1	1	1
	**52**	North G	85	y	8.5	3	0	13	5	26	18	15	3	33	3	2	0	3
	**59**	North G	100		29.5	0	0	3	0	0	1	1	0	0	0	0	0	1
	**77**	North G	100	y	7.9	12	627	48	3	60	39	34	5	66	223	3	2	3
	**114**	North G	75		*n/a*	2	316	13	0	16	15	15	0	21	44	2	1	2
	**131**	North G	100	y	15.7	0	0	13	2	0	41	16	25	70	575	3	2	2
	**50**	South M	75		3.1	0	0	2	0	2	15	15	0	28	1	1	0	0
	**97**	South O	100	y	*n/a*	0	0	1	1	24	14	14	0	6	6	2	0	2
Late	**65**	South Q	100		5.3	2	0	0	0	30	16	13	3	3	4	2	2	3
	**56**	South R	100		4.7	1	0	0	0	0	4	2	2	6	2	1	1	1
	**44**	South S	100		*n/a*	3	0	13	0	0	16	16	0	8	14	3	26	3

This paper is a new analysis of data previously published elsewhere: full details on data collection methods, specimen numbers, and conservation and curation strategies can be found in the cited literature. All materials were analyzed with the consent of the Turkish authorities under the permit from the Ministry of Culture and Tourism, General-Directorate of Cultural Heritage and Museums, provided to the Çatalhöyük Research Project under the direction of Prof. Ian Hodder. Raw data, including dates of recovery, contextual associations, and excavator records, are freely available online at https://www.catalhoyuk.com/research/database.

### Data sets

We base our study on archaeological datasets identified as reasonable correlates of socioeconomic assets. Table 3 outlines these data sets, citing examples of their socioeconomically significant differential distribution in ethnographically documented societies as well as their previous use in archaeological inferences about ancient inequality and summarizing their character at Çatalhöyük specifically.

**Table 3:** Data sets considered in this paper and their inferred relationships to various forms of social differentiation.

	Archaeological criterion	Sample ethnographic, historical, and archaeological studies discussing criterion in relation to inequality	Primarily discussed in relation to	Notes on context at Çatalhöyük
**Subsistence income**	Side room m^2^	[23, 59, 106, 150, 151]	Agricultural income, household production.	Side room space is commonly allotted to storage, particularly of food [75, 76, 93]. We consider side room space an acceptable, if rough, proxy for “land” and /or “food.”
**Productive capacity**	Number of grinding tools with use faces	[53, 103, 152–156]	Productive capacity; relational assets.	Microwear and starch analyses confirm that grinding tools were used for plant—mostly cereal—processing [153, 157].
	Grinding installations use area, cm^2^			
Symbolic elaboration (relational/political assets)	Wall paintings	[45, 70, 89, 109, 121, 158, 159]	Prestige/status, political and symbolic assets.	Many Çatalhöyük houses feature paintings on their walls: monochromatic red panels, geometric designs, handprints, and on rare occasion figurative motifs [160]. Whatever their emic messaging, the paintings clearly carried social messages.
	Bucrania and other animal part installations	[66, 85, 109, 112, 115, 161, 162] *Note: [19, 20, 163, 164] measure hunting success; [19] measures food sharing. We deem this a plausible proxy for either/both*.		Faunal installations—skulls, horns and other animal body parts that are embedded in walls, benches, and other architectural features—are widely considered evidence of social/symbolic prominence [85, 109].
	Number of individuals buried in Building (MNI) Number of primary burials Number of secondary burials	[23, 109, 112, 124, 159]	Ritual assets; relational somatic wealth (embodied human assets)	Subfloor burial was standard practice at Çatalhöyük. As burials were commonly placed in the same locations within houses, many buildings contain complex deposits formed by sequences of individual interments. Both primary and secondary burials are well represented among the remains [82, 165].
	Faunal “special deposits”	[47, 84, 115, 166]	Symbolic, political economic assets	Discrete and contextually or osteologically atypical deposits of animal remains are plausibly linked to ritualized social activities. Examples include bundled aurochs horn cores, groups of minimally processed large animal bones, and bones deposited inside platforms during construction [83, 84, 86, 93, 167, 168].
	Number of grave goods	[66, 100, 147, 161, 169–173]	Symbolic, relational and economic assets	Some items were clearly intentionally placed in graves (e.g., bead strings, rings). Other items discovered in grave fills may or may not have been deliberately placed. We include only items we are sure were intentionally included in burials in our main text discussions. Readers can find the data for all items recovered from grave fills in S3 Table.
	Marine Shells Number of non-local shell specimens Number of locations in which shell taxa originated	[171, 174, 175]	“Costly” or “prestige” goods	Non-local (exotic) marine shells originated tens or hundreds of km from the site; their presence at Çatalhöyük reflects investments of time and energy. They were used to make bodily adornments, suggesting further associations with personal prestige and/or identity [173, 176, 177]. S3 Table includes a breakdown of the shell assemblage into marine (Mediterranean and Aegean) and fossil (Hatay and Taurus) shells.
**“Costly” or “prestige” personal goods**	Non-local stone Number of non-local beads Number of non-local raw materials	[153, 161, 178, 179]	“Costly” or “prestige” goods	At least 30 different types of rocks and minerals are represented in the Çatalhöyük bead assemblage. Some, such as fluorapatite, derive from sources tens or hundreds of km from the site [173]. S3 Table includes the number of stone materials.
***Additional lines of evidence not included in main body of analyses here*: *data available in Supplementary Information***.				
	House size Internal m^2^	[42, 59, 106, 148, 149]	Number of residents (somatic wealth); economic wellbeing; space available for social/relational activity.	While widely used in inequality comparisons, at Çatalhöyük house size (and thus internal m^2^) is constrained by surrounding structures.
	Side room m^2^ / internal m^2^		Comparing storage/production vs. social/ritual asset	The ratio of side room m2 to main room m2, previously used to assess inequality at Çatalhöyük, is potentially affected by house layout constraints. Evidence that main room spaces were sometimes used for storage and food production also renders the significance of the measure unclear.
	Platform m^2^		Symbolic/relational assets	At Çatalhöyük, platforms are strongly associated with burials, installations, and other forms of symbolic/ritual activity [70].
	Number of known house rebuilds	[109, 124, 159]	Symbolic/relational significance of the house	Used to build previous assessments of inequality at Çatalhöyük, but the size of the data set is limited. There are too few fully-excavated sequences of superimposed houses to permit effective comparison.
	Total cm^2^ of grinding tools use faces	[153–156]	Productive capacity	The data track closely with the number of grinding tools. We exclude them from our in-text charts as redundant for discussion and in order to maximize figure legibility.
	Ratio of subadult: adult burials		Productive capacity (size of labor pool); symbolic/relational wealth	Individuals of all ages were interred intramurally. Subadults and adults are represented among both the primary and the secondary interments [82].
	Dietary stable isotopes average ^13^C average ^15^N	[49, 180–183] *Note: [184] discusses isotope values with respect to identity rather than status per se*.	Dietary distinctions; land usufruct (unequal access to food, landscape areas at base of food chain)	Dietary isotope values are shaped by both biological (e.g., age, health) and social influences. As only skeletons with good collagen preservation yielded data, many buildings contained individuals for whom no ^13^C or ^15^N values are available.

Many studies of ancient inequality use overall house sizes as a primary data set [13, 57, cf 97, 146, 147]. House internal area is also a widely-used line of evidence in archaeological discussions of inequality [42, 59, 106, 148, 149]. At Çatalhöyük most houses abutted others on all four sides, constraining house scale and outline. We therefore exclude both house size and internal area from our assessments, although readers can find the data for both in S3 Table. No monumental architecture has been discovered at Neolithic Çatalhöyük, precluding its inclusion in analyses [cf. 13]. As Buildings 114 and 132 lack complete floor plans, they are excluded from architectural comparisons.

We rely on deposits that tie in stratigraphically to specific house constructions, occupations, and abandonments. Many buildings have noteworthy deposits lying in fills well above floor levels, but such deposits are rarely linked to the house shells in which they lie. We include all material that after careful contextual analysis we deem securely linked to particular house use-lives [see papers in 71, 185–187 for details]. Our analyses aggregate data from all biographical phases of each building: foundation, use, and abandonment. We do so primarily for pragmatic reasons, deeming the benefits to sample sizes of such aggregation worth the lowered levels of precision, especially as Çatalhöyük houses were mostly quite short-lived [60, 188] and it is unclear that enhancing the temporal granularity of our analyses would contribute more signal than noise to our inferences.

We turn now to describing each data set’s character as well as recovery at Neolithic Çatalhöyük. We group our data sets according to their posited relevance to various forms of socioeconomic differentiation—productive capacity, staple assets, interpersonal relations—even as we again acknowledge the likelihood that assets originating or expressed in one sphere had value across others as well.

### Economic differentiation

#### Productive capacities: Grinding capacity

Grinding tools form one of the main components of productive technologies at Çatalhöyük. Detailed spatial and contextual analysis suggests that these implements had close associations with houses and formed part of the regular household toolkit [79, 153]. There is currently no evidence to suggest that “cooperative grinding”–that is, multiple grinding toolkits positioned in the same space—was practiced at Neolithic Çatalhöyük [153]. The assemblage includes both portable tools and grinding installations embedded in building floors and platforms [53, 153].

**Fig 3:**
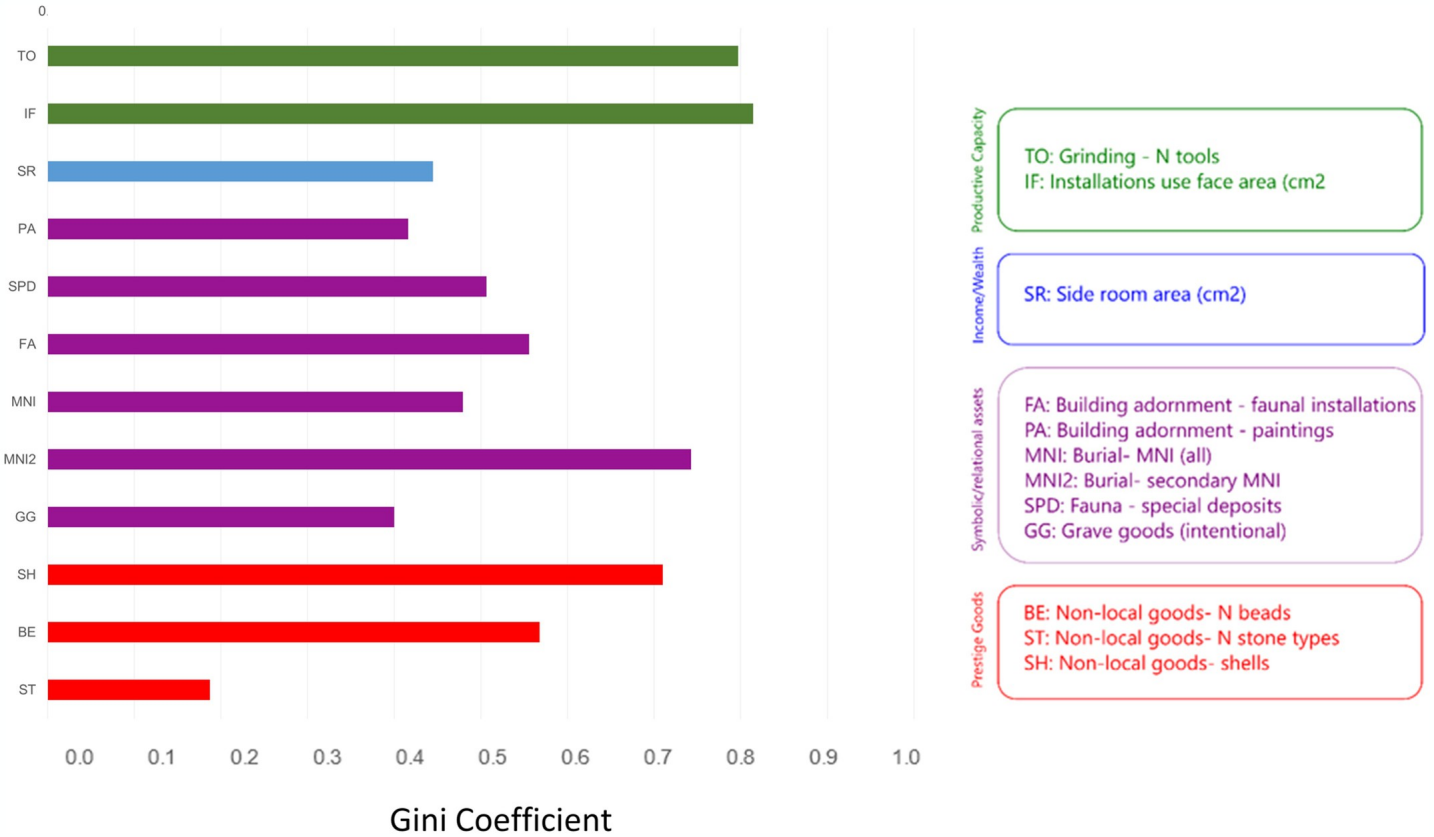
Inequality within a neighborhood. Data derive from nine buildings assigned to the North G occupation at Çatalhöyük.

**Fig 4:**
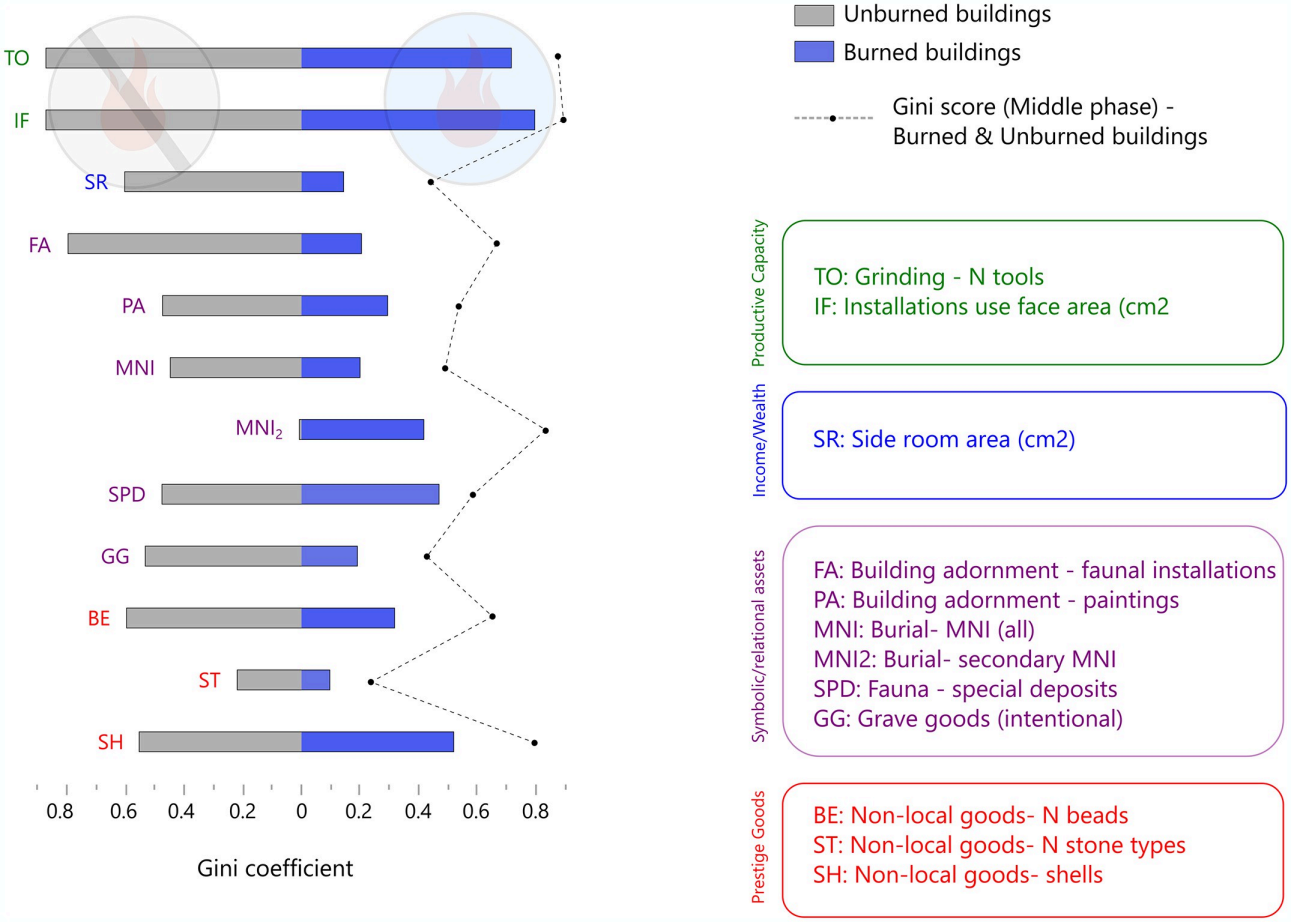
Inequality across middle Çatalhöyük burned and unburned buildings. Same notes as for Fig 3.

**Fig 5:**
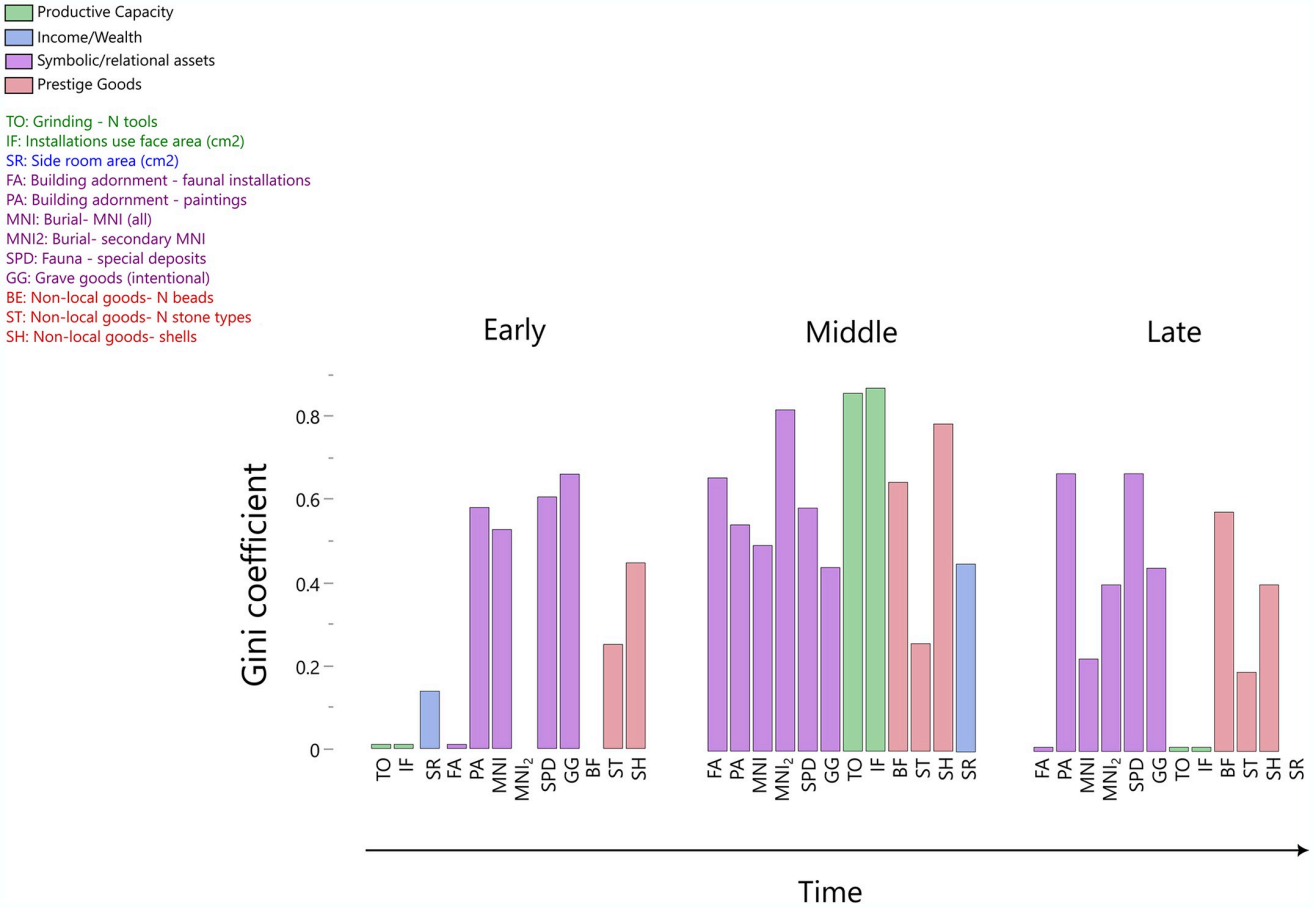
Inequality through time. Buildings are grouped into Early/Middle/Late Çatalhöyük occupations. Side room area (SR) is excluded from the Late histogram due to insufficient data.

Ethnographic research highlights a correlation between the size of the grinding surface area and grinding capacity measured as flour produced per hour [154–156]. Quern size varies considerably at Çatalhöyük, with weights ranging from 2 to 54.6 kg. Our grinding stone data derive from buildings’ occupation and abandonment phases. They thus testify to the actual or—if some stones were placed in buildings as part of abandonment rituals—fictive productive capacities of individual houses [see 80, 93, 95]. We therefore analyze the use area of each house’s grinding installations and tools. We also consider the number of usable implements per building, which plausibly reflects a house’s labor pool. A dearth of heavily used tools and the abandonment of fully functional tools suggest that grinding tools were not curated, heritable implements [79, 153]. Rather, Wright [53], Benz [189], and Benz et al. [190] use inferred deliberate destruction of ground stone tools to argue against wealth/assets being transmissible. The portable tools data are excluded from Figs 3–5 to make them more easily readable; S3–S6 Tables demonstrate that they align almost exactly with the other two lines of evidence.

#### Subsistence income: Household storage capacities

Side rooms were used for storage; they were where food and raw materials were kept in both installed and portable containers [75]. Their areas plausibly reflect the scale of household stores, particularly crops, and as such are widely used as strong proxies for household agricultural production.

### Symbolic/relational differentiation

The lifespan of houses at Çatalhöyük varied, with some standing for a few generations (~80 years), and others for shorter times (~50 years) [191:78]. Variation also exists in their layouts and ornamentation. Some houses are single-room structures, while others were divided into a combination of one “main” room with, ordinarily, one or two “side” rooms [192:41]. Houses do have a broadly modular layout. They typically contain a core series of features: ovens and hearths, storage facilities, and benches and platforms under which consecutive primary burials regularly show evidence of disturbance and sometimes curation of the human remains within. Platforms *tend* to be set against houses’ northern and eastern walls, while ovens are against the south walls and associated with smaller hearth structures, and storage bins are typically placed in side rooms. However, there are frequent exceptions to these patterns and at a granular level there is a remarkable diversity in the morphology and distribution of house features.

Previous work has identified some of Çatalhöyük’s houses as more elaborate than others in terms of organization, ornamentation, and internal furnishings. Such structures ("History Houses" [109] or "Shrines" [66]) potentially acted as social focus points and mechanisms for ‘memory making’ [193]. We do not use the full “Architectural Elaboration Index” developed by Hodder & Pels [109: Architectural Elaboration Index = Floor Segments + Benches + Basins+ Installations+ Pillars + Paintings], as we do not feel confident assigning a particular social or economic significance to buildings’ numbers of floor segments, benches, or pillars; tallying the number of basins in a structure is a complex and uncertain prospect [67]; and we do not exclude the contents of side rooms from consideration. Our quantitative analyses are thus distinct from those previously used to identify History Houses or Large and Elaborate Buildings.

#### Paintings

Some, but not all, of Çatalhöyük’s buildings have paintings on their plastered walls. Such paintings—which range from representational art to handprints to simple swaths of color—presumably reflect one or more kinds of symbolic activity, and their production, modification, and observance surely had social significance. Paintings have also, as previously noted, been central elements of previous discussions of symbolism and social organization at Çatalhöyük [e.g., 66, 81, 109, 112, 194].

Our analyses are based on the data reported in Busacca [160]. We acknowledge Düring’s [110, 123, 127:195] critique that, as Çatalhöyük buildings can have hundreds of plaster layers on their walls, some paintings may have been overlooked during excavation. Fortunately, the houses discussed in this paper were all excavated by skilled—often professional—excavators who were alert to the possibility of wall paintings, and conservators were always on site to support painting identification and recovery.

#### Faunal installations and special deposits

Quotidian faunal remains were removed from most Çatalhöyük buildings at abandonment [134, 195, 196], so the faunal data included here consist of discrete and often deliberately placed faunal deposits. Faunal installations such as skulls and horns embedded in walls, benches, and other architectural features are considered associated with buildings’ occupations only when clearly associated with the use of specific buildings. Many middens and fills contain the remains of dismantled installations, but these are excluded from consideration. Likewise, “special deposits” such as discrete clusters of minimally processed large animal bones and remains deliberately placed in the mouths of bins and ovens are included only when affiliated with specific structures. Such deposits are inferred to be the remains of socially significant events such as feasts, and many appear to have been placed at times that were important in houses’ use-lives [76, 83, 84, 86, 122]. The symbolic and relational significance of animal part installations—the most famous of which are the complete bucrania displayed in a minority of houses—is even clearer, and numerous publications have treated such installations as central to discussions of social organization and symbolic activity at Çatalhöyük [e.g., 63, 66, 85, 109, 197].

#### Burials

ÇHRP excavators have recovered the remains of at least 741 individuals from stratified contexts: 471 from primary burial contexts and 270 from secondary and tertiary contexts [82, 165], almost all from subfloor burials directly associated with specific houses. We do not know how villagers decided which dead would be buried beneath which house: many site archaeologists do not believe that it was purely a matter of lifetime residence. Notably, Hodder & Pels [109] discuss subfloor burials in terms of “symbolic capital,” reflecting particular buildings’ function as ritual nodes within the settlement, or “History Houses”. We tally the number of individuals interred within a structure, considering their postmortem presence as potential assets in terms of both embodied and relational wealth and their (probable) lifetime affiliation a biological asset. House members did not necessarily draw on these assets, but we assume that they could have if they so wished. We provide data on both primary and secondary inhumations, as the two forms of burial may have had divergent socioeconomic implications. Primary inhumations data are excluded from Figs 3–5 to make them more easily readable; all data are provided in S3–S6 Tables.

#### Grave goods

A minority of burials contained material culture (here referred to as “grave goods”), most commonly items of personal adornment such as bone rings and beads. Grave goods are not necessarily associable with individual skeletons, as newer burials frequently cut into older ones and many skeletons are disturbed or disarticulated (some purposefully, in antiquity). We focus on the contents of entire burials, rather than those associated with individual skeletons, as at Çatalhöyük many graves include multiple individuals, and we cannot be sure with which remains the interred grave goods were associated.

Assessing the intentionality of many grave goods’ presence is difficult, as grave fills derive from nearby middens and fills that unsurprisingly often include artifactual and ecofactual “noise.” We therefore conducted three sets of analyses, on (1) grave goods that were clearly intentionally placed in burials, e.g., personal adornments on skeletons; (2) grave goods that were clearly placed plus those that *might* have been placed; and (3) all items recorded as coming from the fill of the grave, opting out of deciding which items are of the greatest significance. Acting conservatively, we present only the first set of analyses in the main text of this paper; the other two are in S3 Table.

#### Personal “costly” or “prestige” goods

Small portable items that could not be acquired locally are here categorized as “costly” or “prestige” goods, as non-local (“exotic”) resources represent higher acquisition costs than local ones. Their size and portability distinguish them from the building-scale assets listed previously, and we consider them suitable for individual expressions of identity and interpersonal engagement. Two data sets are included in this category of non-local materials: shells and stones.

Çatalhöyük’s residents used numerous types of rocks and minerals to make small portable goods. The settlement is located in an alluvial landscape, so key stone materials were procured from significant distances away [78, 79, 153]. For example, bead-makers used not just the limestones and marbles found within a day’s walk from the site (ca. 15 to 20km), but also carnelian that probably came from the Erenler-Alacadağ volcanic formation ca 60–70km away, and fluorapatite and turquoise-colored minerals (azurite, malachite) sourced from areas much further away than that [78, 173].

Çatalhöyük’s non-local shells include both Holocene-dated marine shells and fossil shells. Holocene marine shells include 12 species that originate from the Aegean and the Mediterranean seas and two species (*Antalis dentalis*, *Ostrea edulis*) that live only in the Aegean. These shells thus originated at least 150 (Mediterranean) or 400 (Aegean) km from Çatalhöyük. Fossil shells are primarily *Dentalium*, deriving from the Hatay and İskenderun basins more than 300 km from the site. Other fossil shell taxa originated in the Taurus Mountains at least 50 km away [198]. Non-local shells thus presumably had social value; their acquisition required direct or indirect investments of time and energy. Moreover, most non-local shells appear to have been imported as raw materials or finished artifacts for bodily adornment [78, 173, 176, 199]; their primary role was as visible markers of social distinction. Data used here include finished artifacts, pre-forms, and manufacturing waste from a diverse array of depositional contexts. We focus on shells from burials and other primary contexts; data on shells in secondary or tertiary deposits are summarized in S3 Table. We note that during the Early phase of occupation, the majority of Çatalhöyük’s non-local shells were recovered from middens; lower numbers were found in burials and other domestic contexts. In contrast, during the Middle phase of occupation, the vast majority of non-local shells were recovered in burials and inside buildings. As a result, the numbers of non-local shells *in buildings* changes through time, and inferences about house distinctions that are based on Early-phase shell data must be treated with caution.

#### Other data sets

Some data sets are presented in our supplementary tables but excluded from quantitative comparisons of houses due to either the interpretive complexities of the data set or to our perception of only limited correlation between the data set and socioeconomic variability at Çatalhöyük. These include dietary isotope values, which are not only shaped by myriad social and biological factors but also not equally available for all structures due to taphonomic issues; “grave goods” where the intentionality of placement is unclear; and, as noted previously, building size and internal area. Additionally, some lines of evidence are excluded from quantitative analyses due to sample size limitations (e.g., the number of times a house was rebuilt; the ratio of subadults to adults in subfloor burials). In order to make these data available for colleagues who wish to include them in other studies, we summarize these data sets in S3 Table.

### Methods: Quantitative assessment of inequality

To measure inequality within each data set we use Gini coefficients. We adopt this approach in order to maximize comparability with other studies of inequality in the ancient and modern worlds [39, 57–59]. Gini coefficients measure unevenness in the distribution of observations across a population: unevenness in household storage, for example, or in domestic architectural investment, or in grave goods. Gini coefficients can thus shed light on inequalities of wealth, of health, or of any other sphere of life with diagnostic material concomitants.

Gini coefficients range from 0 to 1. Perfectly uniform distributions (perfect equality across populations) produce Gini coefficients of 0; distributions where a single individual/household/family/etc. controls all assets (over-the-top inequality) produce coefficients of 1. However, Gini coefficients are like averages in that a single number may reflect quite different underlying patterns: the same coefficient could be produced if a settlement dominated by socioeconomically similar households included either a scatter of impoverished or subsidiary households or a cluster of “rich” ones [58, 59]. It is, therefore, important not to end investigation of ancient inequality with the production of a coefficient: one must explore the nature of the underlying data.

We use Gini coefficients calculated for varied data sets as a basis for considering inequality in different cultural spheres. We reiterate that sociopolitical assets are commonly transferable into subsistence goods (“social storage”), and vice versa, so do not intend to communicate that benefits discussed primarily in terms of either sociopolitics or subsistence had value exclusively in either realm. Readers can find all data necessary to explore alternative characterizations of asset value in S3 Table. Please note that in order to maximize the legibility of figures, some data sets are presented only in tables. These table-only data sets have Gini coefficients that match those of other data sets that are included in the figures—for example, the minimum number of individuals in primary burials aligns closely with the number of all interred individuals.

## Results and discussion

### Synchronic inequality

We begin by assessing the evidence for synchronic social differentiation at Çatalhöyük. In Çatalhöyük’s North Area, level North G, researchers have excavated multiple houses with nearly or fully contemporaneous occupations. While these houses existed in close proximity to each other, they had distinct layouts and contents.

Fig 3 indicates that houses displayed unequal levels of ornamentation (paintings and installations), quantities of non-local “prestige” goods, and numbers of people interred beneath their floors. These data suggest that houses within a single neighborhood had pronounced differences in symbolic and relational assets. Also of note is the fact that grinding capacities are dramatically unequal, while side room storage capacities are less unequal but still quite variable. (See S4 Table for the calculated Gini coefficients).

These data are intriguing because they are consistent with economic differentiation that is both significant and socially managed. Kohler and Higgins [106], Kohler, et al. [42], and Bogaard, et al. [151] all found similar differentiation in other food-producing societies, but Çatalhöyük side room area Gini coefficients are at the high end of the range for horticulturalists. If we deem grinding resources an acceptable proxy for productive capacity, and accept Kohler and Higgins’s [106] proposal that storage capacity is a reasonable measure of household income, what we see in level North G at Çatalhöyük is significant inequality in both income and domestic productive capacities. (Bogaard, et al. [151:214] propose that a very high storage Gini at Neolithic Sabi Abyad might be attributable to modern difficulties distinguishing living from storage space. A plethora of evidence associates Çatalhöyük side rooms with storage, but there is certainly evidence of labor in these spaces as well. To the extent that one can differentiate storage space from living space—a complex prospect for any space not blocked from human occupation by installed bins or other storage features—it is reasonable to say that Çatalhöyük side rooms were storage space.) Similarly elevated Gini coefficients are found for remains associated with symbolic and relational wealth. The data are thus consistent with Çatalhöyük’s houses having been both economically and symbolically differentiated during this phase of occupation, but—as noted previously—no data indicate differential access to staple plant foods. While houses’ productive capacities and symbolic assets varied, everyone had access to basic survival resources. This inference is consistent with dietary stable isotopic values from the site: mean human carbon and nitrogen values in adult males and females are virtually identical, and modest inter-individual differences are attributable to age-at-death more than sex [131].

It is possible that the limitations to economic inequality at Çatalhöyük have to do with the specific constraints faced by its farmers. Bogaard and colleagues [11, 151:203–4] explain that societies may increase arable food production by raising labor inputs per unit area (small-scale intensive horticulture), or by expanding the area they cultivate (field agriculture). If people can deploy animal labor in the fields, access to arable land becomes the limiting factor to expanding production; if they cannot or do not rely on animal traction, human labor is generally the limiting factor. Labor-limited farming is broadly associated with relatively low levels of social inequality, whereas land-limited extensive farming aligns with higher levels of inequality [11: Fig 3]. Bogaard and colleagues [11] further propose that urban-scale extensification requires durable inequalities, for harvest-time labor mobilization and maintenance of specialized traction animals. Understanding the roots of such inequalities requires grasping the sources of inequalities in labor-limited economies.

We have no solid evidence for animal traction in Early-Late levels at Çatalhöyük; indeed, it is not until relatively late in the occupation that domestic cattle appear in the assemblages, and when they do it is initially in limited numbers [134, 200, 201]. Botanical evidence further suggests that farming remained labor-limited throughout the occupation [202]. As clear and lasting inequalities linked with agriculture have to do with regimes that promote differential access to land, and as a key ingredient in such regimes is traction, Çatalhöyük’s economy may not have predisposed its inhabitants toward accepting economic inequality across the settlement.

Might the economic variations that we see in fact be attributable not to Neolithic disparities, but to the fact that some of level North G’s buildings burned while full of artifacts and ecofacts, while others, unburned, were emptied at abandonment? Perhaps the North G data reflect differential abandonment practices more than they do lived inequalities. To test whether our perception of inequality is driven by abandonment practices, we turn now to comparing burned and unburned buildings from the Middle phase of the site’s occupation.

### Abandonment practices

Fig 4 reveals far more inequality across buildings that were *not* burned at abandonment than among buildings whose occupations ended in flames. Even more importantly, the Gini coefficients of unburned buildings are broadly comparable to those that we see when we group burned and unburned buildings together. The only data set for which we see dramatically higher values in the combined group than in the separate burned and unburned groups is secondary burials. (See also S5 Table). As Table 2 shows, all of the Middle Çatalhöyük secondary burials in our sample were found in burned buildings, so abandonment practices emphatically do drive their elevated Gini coefficients in the level North G data set. The only other data set with Gini coefficients elevated markedly by grouping unburned and burned buildings together is exotic shells. We note that these shells derive largely from graves, and thus—like secondary interments—are recovered from underneath house floors.

We infer therefore that differential abandonment practices are not primarily responsible for our perceptions of inequality across the site. Instead, Gini coefficients indicate significant inequality across numerous data sets in unburned buildings. They reveal differences in productive capacity across burned ones as well, but very little differentiation in symbolic or relational wealth—even in subfloor, pre-abandonment deposits such as interred individuals and grave goods. The dramatic differences in productive capacity across the burned buildings, moreover, are surely driven by the remarkable quantities of grinding stones placed inside burned Building 77, potentially as part of ritual building closure. Burned buildings look remarkably equal in terms of their diverse assets; unburned buildings do not.

The causes and social significance(s) of structural fires at Çatalhöyük have been a recurrent topic of conversation among site researchers, who over the years have inferred varied forms of incineration and abandonment [93, 94, 96, 203, 204]. Core to the debates has been the issue of whether or not burned buildings were staged for the incinerations, their contents deliberately placed and thus only partially or minimally reflective of the building prior use and occupancy. The evidence for staging is strongest in Building 77, where excavators found multiple collections of valuable and/or unusual material on the building floor: a dog cranium and an eagle claw, heaps of antler, the largest querns yet found at the site (still in usable condition), concentrations of deliberately broken stone tools, and more [95, 205]. These collections, plus large cattle bones in the otherwise sterile burned fill of the building, lead many of the site’s archaeologists to infer ritualized placement of items within the building, perhaps followed by further, commemorative, deposits later [80, although see 94 for a degree of caution, 125]. Tsoraki [80] further suggests that the sheer quantity of grinding stones placed inside Building 77, in addition to their varying wear stages, may reflect contributions from multiple households, consistent with house closure (and, by extension, potentially those of other houses, as per [83]) being a communal event. Burned Buildings 52, 80, and 131 also contained exceptionally rich and varied material culture, some or all of which might have been intentionally placed [93, 134, 204].

The Gini coefficients indicating mostly *but not entirely* low inequality coefficients across burned buildings are consistent with burned buildings being “special” but not in an entirely unified fashion. Grave goods, for example, are unequally distributed across structures in the Middle phase of the occupation (Fig 5, S6 Table), but they are quite equally distributed across burned buildings (Fig 4). Burial MNIs and primary interments (Table 2) follow a similar pattern: they are unequally distributed across all Middle buildings, and relatively equal across burned buildings only. As subfloor burials and grave goods should—unlike faunal or grinding stone installations—remain unaltered by abandonment practices, the rough equality across burned buildings might reflect broadly similar levels of symbolic or relational “wealth” among houses abandoned in a distinctive fashion. That this parity is meaningful might be supported by the fact that unburned buildings are markedly different from burned ones with respect specifically to the subfloor categories of secondary burials and exotic shells. Readers should be cautious not to place much weight on this inference, however, given the small number of burned buildings included in these analyses. It is possible that buildings were selected for burning on the basis of their relational or symbolic positioning, but it is also possible that, as Farid [96] posited, Çatalhöyük’s buildings burned in different ways and for varying reasons (e.g., ritual closure, reprisal arson [206], accidental fire spread).

Finally, the contrast between burned and unburned buildings may, in part, speak to the fluidity of households at Neolithic Çatalhöyük. Burned buildings represent much clearer and more restricted focal points than do unburned buildings; perhaps the variability we perceive among the latter reflects their assorted and mutable relationships to larger social networks. We highlight here, as well, the unequal numbers of *secondary* deposits, which might reflect participation in networks extending beyond the confines of the site itself.

#### Inequality in diachronic perspective

Previous work has posited a peak in social complexity during the middle portion of Çatalhöyük’s occupation, followed by a decline during its Late phase [126, 207]. Çatalhöyük’s population peaked in the Middle phase of its occupation: architecture and bioarchaeological data are consistent with increased fertility fueling a rise in population during this period, followed by population dispersal in the Late period [208, 209]. Given that increases in population *density* would be expected to have biological correlates such as rising disease loads, it is possible that density did not increase along with population size. Villagers seem to have remained reasonably well-nourished throughout, and the adequacy of their diets may have mitigated the physiological stresses of life in a crowded environment [208, 209]. However, site bioarchaeologists do infer more (potentially relevant) interpersonal violence, as well as higher workloads, in the Middle phase than either earlier or later phases [165, 206].

Did population growth contribute to rising inequality or social stratification, at Çatalhöyük? It would not automatically have triggered hierarchization, but it could have accelerated or contributed to its development. Alfani [58:30] uses a wide range of prehistoric and historical evidence to argue that “In preindustrial times, there was no *necessary* cause of inequality growth…There were instead a number of *sufficient* causes of inequality growth, among which we could name economic growth, demographic factors …, and so on. When one or more of these potential causes became active, inequality grew.”

Our inferences must be considered tentative, as available Early phase data derive from four buildings, three of them 75% excavated, while our Late sample consists of three fully excavated buildings, all of which belong to a single house sequence (B.65-56-44). Our Middle phase sample includes 13 buildings, of which eight have been fully excavated. Inferences about diachronic shifts in inequality can therefore be considered plausible but not certain: this caveat logically applies to non-quantitative studies of diachronic inequality at the site as well, both ours and others’.

Fig 5 suggests that overall levels of difference were highest in the Middle period of the site occupation, but distinctions existed earlier and later as well (see also S6 Table). Furthermore, we see no shared trajectory of inequality across all data sets. A greater number of Gini coefficients are consistent with inequality during the Middle than during the Early or Late phases of occupation, but different data set Gini coefficients rise and fall in different patterns.

Key evidence of differentiation that comes without a caveat due to small sample size dates to the Middle period, where we see clear inequality in side room area. No such differentiation exists with respect to main room area. The agglomerated nature of housing at Çatalhöyük surely constrained overall house sizes, so we do not assume that residents could express wealth by building houses to whatever size they preferred. We nonetheless note—in relation to the argument that side room space is plausibly directly linked to agricultural production and thus to household income, whereas overall house size is a better measure of wealth—that architectural plans at Çatalhöyük can be read as indicating a high level of income inequality, dramatically tamped down when it comes to measures of wealth [e.g., 210:133].

Such tamping down might explain, at least partially, the drop in mortuary differentiation (grave goods) from the Early into the Middle phase of occupation. This drop is very noticeable in Fig 5, which reveals rising differentiation in multiple other data sets at this time. Productive capacities (food processing), which were equitably distributed during the Early phase, become extremely unequally distributed during the Middle phase. So do symbolic assets (faunal installations). Prestige goods (shells), unequally distributed during the Early phase, become even more so during the Middle phase. The flattening of the burial Gini coefficients during the Middle phase plausibly reflects active—and perhaps conscious—efforts to keep rising disparities in income from contributing to, or even institutionalizing, other distinctions between members of society [although see 81]. However, the peaks in installations and shell Ginis suggest that such attempts were only partially successful, and that some houses had greater access to symbolically and/or politically valuable goods. These findings support Mazzucato’s [88] network analysis, which identified an increase in social connectivity from the Early to the Middle occupations that developed specifically through the mediation of a group of elaborate and highly interconnected buildings.

We previously noted that botanical and faunal data from Çatalhöyük are consistent with a labor-limited farming economy, and that human bioarchaeologists infer peak physical labor during the Middle occupation levels. Perhaps labor mobilization was particularly important to the residents of Çatalhöyük during this phase, which is also characterized by peak distinctions in grinding and domestic storage capacities. Economic inequalities that are rooted in differential success in mobilizing labor are hard to maintain, however, and from the Middle to the Late phase of occupation sharp drops in Gini coefficients characterize many data sets, most dramatically house grinding capacity. These data broadly align with existing inferences about population dispersal and declining complexity in the Late period. Neither wall paintings nor faunal special deposits became more equally distributed, however, and shell Gini coefficients also remain high. All of these data sets can reasonably be interpreted as symbolically laden, so we might infer that ritual differentiation remained in place even as economic differences decreased. Such an inference is at present mostly speculation, though, as the three Late houses are closely related structures, built directly atop each other and cited in other publications as belonging to a single house “sequence” [e.g., 109]. Future excavations may well reveal considerably more variation across the settlement during its Late phase of occupation.

#### Exploring different kinds of inequality, and the relationships among them

The Çatalhöyük data are consistent with significant socioeconomic variability but not with concentration of staple resources or critical capabilities [sensu 152]. We see a broad accordance with Crumley’s [211–213:144] definition of heterarchy: a social system in which each component element “possesses the potential of being unranked (relative to other elements) or ranked in a number of different ways….” Myriad rankings exist, but they are not mutually consistent or temporally stable, and the social structure is not pyramidal. This definition fits the Çatalhöyük data well. It does not, however, shed extensive light on sociopolitics in and around the ancient village, as heterarchies encompass varying degrees of stratification [214]. As Rosenberg and Rocek [45:19] note, “heterarchy” functions in many ways as “a remainder category for hierarchies that do not fit the categorical (i.e., pyramidal) archetype. … simply noting the presence of inequality in some communities or calling the organization of some communities heterarchical conveys as little meaningful information as calling a group at the other end of the organizational spectrum egalitarian, and ultimately requires us to provide some level of detail concerning the actual organization of the groups being discussed….” We follow, therefore, Rosenberg and Rocek [45] in embracing McGuire and Saitta’s [215] non-typological complex communal society model, which accommodates both egalitarianism and hierarchy “depending on circumstances and where in a given socio-political system one chooses to look.” This approach, which *expects* intrasocietal variability and dynamic fluidity, encourages us to consider the specific elements of egalitarianism, distinction and hierarchy in various aspects of our data set.

It is a truism that inequality may not develop—or be expressed—similarly across different social spheres. Drennan, et al. [216:71], for example, explore “dimensions of variability” in inequality, not just a single/linear axis of development, insisting that archaeologists must *posit* relationships between different socioeconomic dimensions for evaluation (not just assume that if A is unequal then B must be as well)—and consider diachronic change. Several scholars have clarified that wealth inequality need not align with inequalities in production or in consumption, or with social and political hierarchies [e.g., 27, 59]. That said, of course inequalities may travel together, with one form of inequality facilitating or enhancing another. If redistribution is not the norm, inequalities in production presumably lead to wealth disparities; unequal wealth may mean unequal opportunities to host feasts vital to the accumulation of political power. Multiple material culture inequalities may also share a root cause: unequal production could, for example, lead to both unequal diets and unequal access to prestige goods.

Our data indicate dramatic differences in domestic productive capacity during the Middle phase of occupation at Çatalhöyük. Differences in household “income”—inferred on the basis of storage capacity—are smaller, although they still hover at the upper end of those recorded in labor-limited farming economies [11]. We thus infer meaningful economic differentiation at Çatalhöyük during its Middle phase of occupation. These economic distinctions did not carry across into differential access to the basics of survival, however: all well-sampled houses have yielded evidence for suites of cereals, pulses and fruits/nuts. Economic differentiation may well have been lower during the Early and Late phases of occupation at the site, but as yet few houses dating to either time period have been fully excavated.

Middle Çatalhöyük houses also varied with respect to their symbolic assets and prestige goods. As most of the data sets interpreted here as primarily associated with interpersonal relations (wall paintings, faunal installations, burials) are about as unequally distributed as domestic storage capacities, we infer that symbolic and relational inequality existed at roughly the same level as income inequality. However, we do not see correlations between high incomes or productive capacities and symbolic or relational wealth. As Tables 4 and 5 reveal, some buildings with high storage capacities (inferred “income”) have few or no burials or installations (Building 59). Some are rich in both storage capacity and interments (Building 131). Buildings that have below-average storage capacities may be phenomenally symbolically wealthy (Building 77), or unspectacular (Building 3). The two buildings with the highest inferred productive capacities (grinding tools) have very different quantities of symbolic assets (Buildings 114 and 77). Costly portable goods (non-local shells and stones) are found in houses with both above- and below-average storage capacities.

**Table 4:** Comparison of assets associated with middle Çatalhöyük phase houses. Assets are color-coded on a continuum from green (highest) to red (lowest) values.

Building	Level	% of building that was excavated	Burned building?	Side room m^2^	# of grinding tools with use faces	fixed grinding installations = area of use face in cm^2^	Paintings	Faunal Installations (in situ)		Faunal special deposits	Burial MNIs (primary and secondary)	# of primary individuals	# of secondary individuals	Grave goods (intentionally placed)	Exotic shells	# of exotic shell sources	# of exotic stone beads
**132**	North F	75		*n/a*	0	0	3		0	0	10	10	0	29	6	2	1
**114**	North G	75		*n/a*	2	316	13		0	16	15	15	0	21	44	2	1
**97**	South O	100	y	*n/a*	0	0	1		1	24	14	14	0	6	6	2	0
**59**	North G	100		29.5	0	0	3		0	0	1	1	0	0	0	0	0
**131**	North G	100	y	15.7	0	0	13		2	0	41	16	25	70	575	3	2
**1**	North G	100	y	9.8	0	0	21		2	15	60	52	8	72	85	2	3
**52**	North G	85	y	8.5	3	0	13		5	26	18	15	3	33	3	2	0
**77**	North G	100	y	7.9	12	627	48		3	60	39	34	5	66	223	3	2
**3**	North G	100		6.3	0	0	15		2	30	8	8	0	20	6	2	0
**119**	North F	75		6	0	0	2		0	0	0	0	0	0	0	0	0
**50**	South M	75		3.1	0	0	2		0	2	15	15	0	28	1	1	0
**49**	North G	100		2.3	0	0	31		0	25	15	15	0	38	54	2	0
**51**	North G	100		0	0	0	0		0	0	0	0	0	0	3	1	1
***Average***				*8*.*9*	*1*.*3*	*72*.*5*	*12*.*7*		*1*.*2*	*15*.*2*	*18*.*2*	*15*.*0*	*3*.*2*	*29*.*5*	*77*.*4*	*1*.*7*	*0*.*8*

**Table 5:** Distribution of assets across a “neighborhood” (Hodder level North G). Above-average numbers are boldfaced.

Building	Level	% of building that was excavated	Burned building?	Side room m^2^	Number of grinding tools with use faces	Total area of use face of grinding tools (cm^2)^	fixed grinding installations = area of use face in cm^2^	Paintings	Faunal Installations (in situ)	Faunal special deposits	Burial MNIs (primary and secondary)	# of primary individuals	# of secondary individuals	Grave goods (intentionally placed)	Exotic shells
**1**	North G	100	Y	9.8	0	0	0	**21**	**2**	15	**60**	**52**	**8**	**72**	85
**3**	North G	100		6.3	0	0	0	15	**2**	**30**	8	8	0	20	6
**49**	North G	100		2.3	0	0	0	**31**	0	**25**	15	15	0	**38**	54
**51**	North G	100		0	0	0	0	0	0	0	0	0	0	0	3
**52**	North G	85	y	8.5	3	**3710**	0	13	**5**	**26**	18	15	3	33	3
**59**	North G	100		**29.5**	0	0	0	3	0	0	1	1	0	0	0
**77**	North G	100	y	7.9	**12**	**9453**	**627**	**48**	**3**	**60**	**39**	**34**	**5**	**66**	**223**
**114**	North G	75			2	**495**	**316**	13	0	16	15	15	0	21	44
**131**	North G	100	y	**15.7**	0	0	0	13	**2**	0	**41**	16	**25**	**70**	**575**
*Average*				*10*	*2*	*1518*	*105*	*17*	*1*.*56*	*19*.*1*	*22*	*17*	*5*	*36*	*110*

In contrast to economic assets, the small samples of Early and Late houses suggest inequitable distribution of symbolic/relational resources. When we look at the Çatalhöyük data by phase of occupation, we see that Gini coefficients are consistently high for faunal special deposits, wall paintings, and exotic shells (although we remind readers that Early Çatalhöyük shells derive largely from middens instead of buildings, so caution is warranted). Table 4 shows no inverse correlation between paintings and faunal remains, so we do not believe that these Gini coefficients reflect some villagers having chosen to adorn their buildings with paint and others preferring animal remains.

Meanwhile, the Gini coefficients for burial MNIs and grave goods both decline through time. Rather than a decline in overall social differentiation, the declines in mortuary inequality may reflect increasing standardization of mortuary practices, perhaps contributing to social cohesion in an increasingly large settlement. (See [217] for deeper discussion of the potential levelling role of mortuary practices.) It has often been proposed that southwest Asian Neolithic societies placed social constraints on visible signifiers of social distinction, with inequities (usually visualized as economic in origin) being masked or suppressed through leveling or integrative practices such as mortuary ritual or feasting [e.g., 46, 47]. Our data are consistent with economic inequalities in the Middle phase of occupation. It is plausible that mortuary traditions helped foster a sense of community in the face of economically induced tensions.

Are there other lines of evidence that could support the idea that Çatalhöyük’s residents performed equality? Other models of social organization at Çatalhöyük [e.g., 109] contrast houses rich in “history” with economically productive ones, with the latter provisioning the ritually and politically powerful former. In contrast, our data reveal that while economic and social assets did not necessarily travel together, neither were they separated into discrete buildings. Our findings further indicate that burned buildings were dramatically distinguished from unburned—but among themselves, not heavily differentiated, at least in quantitative terms. We might, then, infer a performed difference between comparatively standardized “special” and diverse “non-special” places. This is plausibly again consistent with performative flattening of quotidian social distinctions. That building fires would have had dramatic economic ramifications, and that they occurred during the phase of occupation when Çatalhöyük’s population and levels of intracommunity violence both peaked, arguably provide further support for the idea that site residents were performing equality in the face of social tensions. Differences across houses in symbolic and prestige assets complicate this inference, however: Çatalhöyük’s story was not simply one of agriculturally induced inequality ameliorated or obscured by ritual activity.

### Living unequally at Çatalhöyük

Having identified broad patterns of material differentiation across Neolithic Çatalhöyük, the next goal is to look at how these difference played out in the social lives of individuals living at the settlement.

Our data, which suggest no clear relationships between economic and social/symbolic assets, complicate specific elements of previous inferences about how Çatalhöyük’s residents established and negotiated economically and socially varied positions. Houses that are full of symbolic elements presumably played crucial roles in the ritual life of the community [as per 109], but as most such houses have moderate storage, and their productive capacities vary, our data do not support that such houses helped to manage and transfer productive success into symbolic realms. Looked at more broadly, however, our data align with Hodder and Pels’s [109] inferences that at Çatalhöyük houses had varying degrees or forms of involvement in ritual, and that prominence in the ritual and political sphere did not derive in any straightforward manner from economic success. Our findings also align with the big picture provided by Wright [53], who, in examining house-by-house lithic artifact inventories, identified deliberate non-transmission of valuable ground stones in some but not all houses: surely evidence of a complex attitude towards material wealth and inheritance. Our findings, like hers, suggest a society grappling with the tensions between maintaining egalitarian norms and evolving social complexities.

More granular analysis of individual households can and should yield insights into the nuanced dynamics of inequality within this ancient community. The key is the way in which we interpret the material culture itself. Understanding fluid and intangible social inequalities necessitates examining the *habitus* of interactions in various contexts, such as food preparation and communal feasting. Doing so is possible to a degree [76, 122, 218, 219], with data from inside structures complemented by external faunal and botanical evidence as well as the occasional presence of communal ovens [e.g., in Space 333; 220].

**Fig 6:**
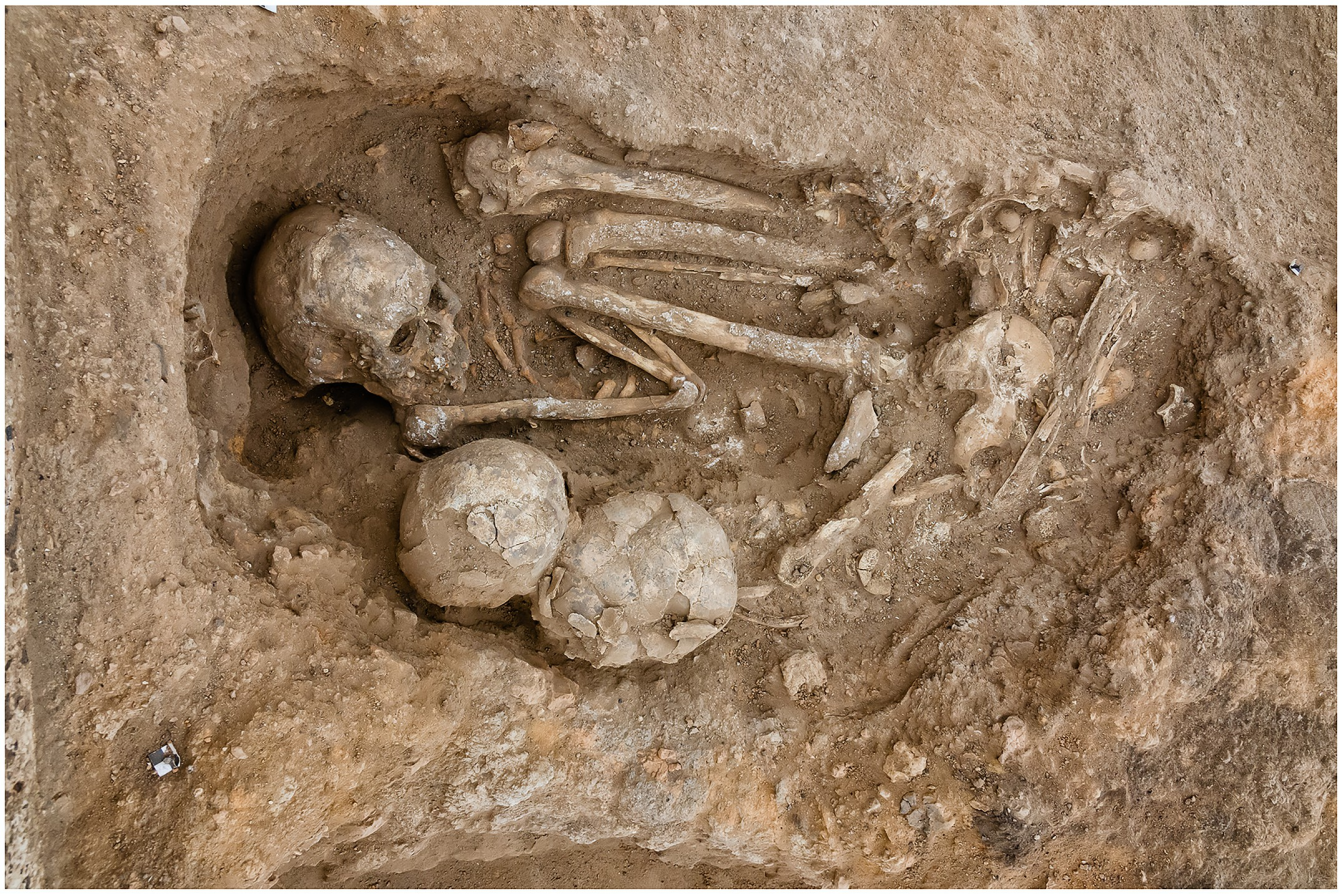
Late Çatalhöyük (6500–6300 BC) burial feature F3684 from building 129. The remains of an adult male are accompanied by two additional crania.

Beyond this, much has been made of the ways in which daily practice and social memory manifest in the archaeological record at the site [221], but recent analysis of funerary practice, focusing on the curation of bodies both prior to interment as well as secondary interment, and subsequent curation of human remains, hints at another potential area where social interactions and inequalities may have been negotiated. Some of the burial sequences demonstrate evidence of having been re-visited to remove or re-organize skeletal remains, and in a minority of cases to remove or re-deposit crania or crania and mandibles (e.g. "skulls") [222]. These secondary deposits of human remains often occur in association with primary inhumations, which suggest some type of social distinction shared with some but not all individuals. An example occurs in the Late phase (ca. 6500–6300 BCE) burial feature F3684 from Building 129, where the remains of an adult male are accompanied by two additional crania along with a large amount of disarticulated infracranial remains (Fig 6). One of the most elaborate secondary burials occurs in Middle Çatalhöyük (ca. 6700–6500 BCE) Building 5 in which the remains of a basket containing a cranium and mandible (the frontal bone covered in cinnabar) and accompanied by two finely worked obsidian projectile points, and a shell containing cinnabar pigment were found. Outside of the basket, positioned posterior to the cranium, was a concentration of objects, including four obsidian projectiles, one finely spiral-grooved ground stone mace head crafted from white marble. The obsidian projectile points show no use wear, which indicates that they may have been fashioned solely to be deposited with these remains. Directly above this assemblage was placed above a bundle containing a partially articulated spinal column and limb bones (perhaps from the same individual as the cranium and mandible). The elaborate preparation of the human remains and associated objects are commensurate with an increased energy expenditure associated with the burial of this individual and reflective of social distinction [223].

In terms of the material culture itself, there is a general consistency in the distribution of most types of artifacts and ecofacts across various contexts, including grave goods. While we see little tangible evidence for social interaction across houses embedded within these physical patterns of distribution (such as, for example, ceramic refits, makers marks, or aesthetic or stylistic signatures), there has been discussion that suggest complex social interactions may be implicitly linked to the technological practices evidenced by the material culture, whether through the (communal?) provenancing of raw materials, or in the knowledge and associated craft linked to production of tools [74]. For example, it has been noted that the uniformity in the production and use of flint daggers across different buildings hints at a shared technological and aesthetic culture that transcended individual households [126]. Similarly, examination of obsidian usage at Çatalhöyük illuminates the material’s pivotal role not just in everyday utility but also in reinforcing social stratifications through its symbolic and economic value. It has been suggested that obsidian’s integral place in rituals and burials reflects nuanced social interactions, where resource access and control could have delineated social hierarchies and power dynamics within the community [125].

The technological consistency reflected in the morphology of chipped stone tools suggests an intricate weave of social interactions and collective identities, subtly reflecting an underlying social cohesion or hierarchy within the community. Such shared practices point to intangible social structures, evidenced through common material technologies, that might have played a role in both unifying and differentiating the social strata within the Neolithic settlement. Detailed analysis of, in particular, obsidian distribution and crafting practices reveals a complex socio-economic landscape, where technology and material culture intertwine with social inequality, displaying how prestige and status may have been mediated through controlled access to essential materials.

It is crucial to recognize the limitations of our dataset, which primarily captures ground-level activities within the settlement. Offsite interactions associated with activities such as hunting, the sourcing of raw materials, or the seasonal management of crops, remain largely conjectural, based on the general implications of archaeological findings. In addition, close excavation of some unusually well-preserved burned structures in the last decade of the project (*i*.*e*. Building 89 or Building 79), highlight the likelihood that many of the complex interactions among households occurred in spaces that are not manifest in the main (and ‘normal’) archaeological record of the site. Roofs, for example, are rarely preserved: when they are, evidence such as pyrotechnic installations, walls of lightweight structures and even grinding installations hint at a plethora of complex activities and structures taking place above the private lower footprint of the buildings [224]. If we contrast this dynamic rooftop scenario with the static sterility of the ground-level structures subjected to a ‘conventional’ deconstruction at the end of their use-lives [225], it is clear that the scarcity of roof remains leaves a vital sphere of lived interactions invisible. This makes it challenging to discern the exact nature of inter-household interactions, which clearly did not happen through the walls (despite the presence of occasional crawl holes [226]. These are by no means the norm but, where present, may imply increased social interaction between the residents of the linked buildings). We also know that midden areas, which yield frequent evidence of activity and single-use fire-spots, were dynamic inter-building spaces, which again, likely played a significant role in everyday interactions as spaces where people would have been able to come together [227, 228], even if it remains difficult to piece together exactly how these communal areas and activities might have embodied socioeconomic inequalities.

## Conclusion

This study enriches our understanding of social differentiation in Neolithic southwest Asia and contributes to broader discussions of inequality and early agropastoralism. Integrating multiple lines of evidence enables us to explore the complexity and fluidity of social differentiation across centuries of occupation at Neolithic Çatalhöyük. Our data reinforce current thinking about the complexity of the relationship between food production and economic inequality; they enrich conversations about house closures and ritual practices [e.g., 93].

Nonetheless, questions remain about inequality at Çatalhöyük. We note, for example, that the site’s houses were not static entities. They were, on average, occupied for a few decades, and residents modified their homes to suit their changing needs and situations; only a minority of the resulting structures remained unaltered for the durations of their occupations. Examining patterns of inequality across the occupations of individual houses will enrich the inferences here, and may reveal intriguing trends with respect to symbolic enrichment or economic wellbeing over the course of individual houses’ occupations. Further light may also be shed on the durability of inequality at Çatalhöyük, whether or not assets transmitted across generations, by investigating the biographies of houses in the site’s well-known “house sequences.” Structures in these sequences are built one atop the other, each new house’s walls are founded on the footprint of its predecessor; presumably, the superimposed buildings’ shared layouts and locations reflect social continuity through time. Comparing and contrasting the developmental characteristics of each of these buildings will illuminate the extent to which they resemble each other symbolically and economically as well as structurally.

## Supporting information

S1 Table Botanical data by area and building, Neolithic Çatalhöyük. The number of ‘samples’ representing each building can be considered as the number of independent behavioral episodes or events [132, 228]. Rather than simply reporting the raw numbers of seed, chaff, nutshell etc. items per sample, occurrence is graded as follows: ‘x’ = trace amounts (<30) within any individual sample; ‘xx’ = at least 30 items within any individual sample and ‘S’ = visible, normally pure storage concentration (associated almost entirely with burned buildings). In the context of recent discussion of the quantitative criteria suitable for identifying deliberate collection of a species [229], it is clear quantitatively as well as stratigraphically/contextually that the ‘S’ occurrences provide the clearest evidence. The distinction between low numbers per sample (‘x’ occurrences) and higher numbers (‘xx’) is arbitrary, like other thresholds that have similarly been used to distinguish chance occurrences from deliberate behavior (e.g. [229] which uses 50 items) and is intended simply to illustrate stark patterns of absolute abundance among plant categories.
(XLSX)

S2 Table Botanical remains in six well-preserved and fully exposed burned buildings. This table summarizes the plant contents of all of the well-preserved and fully exposed burned buildings with visible—and hence presumably intentional—plant concentrations. It corroborates widespread use of all three cereals, at least one pulse crop and at least one other taxon (often wild mustard, in addition to other fruit/nut taxa). H = “History house” [sensu 86, 109]. LE = “large and elaborate” building [110]. “f-t” means “free-threshing.” “x” = <30 items max per individual unit. “xx” = >30 items. “S” = discrete concentration (“storage” deposit). “Ext” = external. Building 150 is not otherwise included in this paper; it is used here only to illustrate a pattern across burned buildings.
(XLSX)

S3 Table Data by building, Neolithic Çatalhöyük. *Holocene marine shells include twelve species that originate from the Aegean and the Mediterranean seas and two species (*Antalis dentalis*, *Ostrea edulis*) that live only in the Aegean. These shells thus originated at least 150 (Mediterranean) or 400 (Aegean) km from Çatalhöyük. The most commonly encountered fossil shell is *Dentalium* (n = 188), which originated in the Hatay and İskenderun basins >300–400 km from the site. Other fossil shell taxa originated in the Taurus Mountains, at least 50 km away [198]. ^ Carbon and nitrogen stable isotope analyses have demonstrated that Çatalhöyük inhabitants consumed animals that had fed in a range of locations across the Konya Plain; intraspecific variation is high and changes through time [131, 133, 230]. Sitewide human C and N data reveal no evidence for sex-based distinctions in diet [131]. There are, however, age-based distinctions in diet [231]. We therefore note the average ages of the individuals providing the stable isotope data in our samples for each house. The sex composition of each sample is also reported. While all unburned, mostly complete primary interments were sampled for stable C and N isotope analysis, only those with good collagen preservation yielded data. Most buildings contained individuals unrepresented in S2 Table data.
(XLSX)

S4 Table Gini coefficients for buildings within a single neighborhood. (XLSX)

S5 Table Gini coefficients for burned vs. unburned buildings. (XLSX)

S6 Table Gini coefficients by phase of occupation. (XLSX)
